# Alkali chalcogenides-assisted vapor–liquid–solid growth of WX_2_ (X = S, Se, Te)

**DOI:** 10.1186/s11671-025-04340-5

**Published:** 2025-09-10

**Authors:** Yi-Cheng Chiang, Po-Yen Liu, Erh-Chen Lin, Sheng-Hung Fan, Chih-Chieh Hung, Yu-Hsiang Cheng, Yi-Hsien Lee

**Affiliations:** Department of Materials Science and Engineering, National Tsing Hua University, Hsinchu, 30013 Taiwan

**Keywords:** Promoter-assisted CVD, Transition metal dichalcogenides, WTe_2_, Ultraclean manipulation, STM, Alkali chalcogenides

## Abstract

Promoter-assisted chemical vapor deposition (CVD) has emerged as a robust strategy for the low-temperature synthesis of diverse transition metal dichalcogenides (TMDs). In these processes, promoter-induced intermediates facilitate specific reaction pathways, enabling controlled growth via vapor–solid–solid (VSS) or vapor–liquid–solid (VLS) modes. While previous studies have primarily focused on transition metal precursors, growth pathways involving engineered chalcogen-based intermediates remain underexplored due to their volatility and low melting points. Here, we demonstrate a stabilized chalcogen strategy that enables the scalable growth of highly crystalline tungsten-based (W-) TMDs through the formation of alkali–chalcogen mixtures within the VLS regime. Atomically resolved scanning tunneling microscopy (STM) of transferred WTe_2_ confirms ultraclean surfaces, attributed to the salt-like alkali–chalcogen interfacial layer that enables support-free film delamination. This work demonstrates a versatile route toward the scalable synthesis and clean manipulation of high-quality TMD.

**Graphical abstract:**

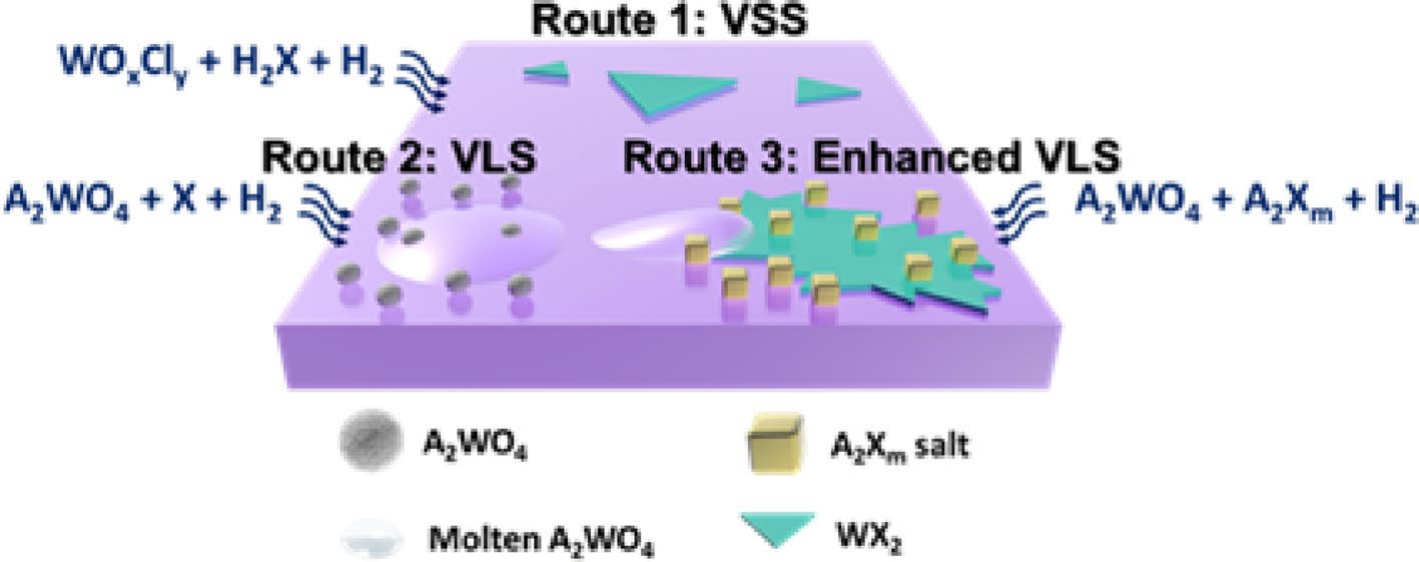

**Supplementary Information:**

The online version contains supplementary material available at 10.1186/s11671-025-04340-5.

## Introduction

Scalable synthesis and clean manipulation of highly crystalline transition metal dichalcogenides (TMD) are essential for both fundamental research and applications. These goals have been realized through promoter-assisted chemical vapor deposition (CVD) methods [1–7]. Among them, alkali halide-assisted CVD received considerable attention, as such promoters enable the growth of diverse TMD materials at significantly reduced temperatures [1–6]. In these processes, the intermediate reactants steer specific reaction pathways, allowing precise control over the growth mechanism, including vapor–solid-solid (VSS) and vapor–liquid-solid (VLS) modes [1–17]. While most growth studies have focused on the roles of promoters and transition metal precursors, growth pathways involving engineered chalcogen-based intermediates remain largely unexplored, due to their low melting points and volatile nature. Contamination-free manipulation of synthetic TMD is critical for probing quantum phenomena. Conventional transfer methods typically involve polymer support layers, such as poly(methyl methacrylate) (PMMA) [14, 18], polydimethylsiloxane (PDMS) [19], polystyrene (PS) [20], or cellulose acetate (CA) [21], combined with chemical etchants (e.g., KOH, NaOH, or HF) [22]. To mitigate film degradation induced by etching, several etchant-free approaches have been proposed, including bubble transfer [23] and salt-assisted delamination [24]. Nevertheless, polymer residues and process-induced damage remain significant obstacles, often compromising the structural and electronic integrity of the transferred films. Development of a residue-free manipulation strategy for synthetic TMD is highly desirable.

Within the family of W-TMD (WX_2_, where X = S, Se, or Te), WS₂ is known for its strong spin–orbit coupling, while WSe_2_ exhibits distinctive p-type semiconducting behavior. WTe_2_, in contrast, is classified as a type-II Weyl semimetal and hosts a range of emergent quantum phenomena, including the quantum spin Hall effect, and interface ferroelectricity [25–32]. Despite their promising properties, the synthesis of W-TMDs remains challenging due to the high sublimation temperature of tungsten oxide (WO_3_) [33]. Here, we study alternative growth pathways for W-TMDs enabled by the stabilization of chalcogen sources via alkali-mediated reactions. Universal growth of highly crystalline W-based TMDs is demonstrated through the formation of alkali-chalcogen intermediates within the VLS regime. Furthermore, atomically resolved scanning tunneling microscopy (STM) of the transferred WTe_2_ films confirms the ultraclean manipulation strategy.

## Promoter-assisted growth modes

**Fig. 1:**
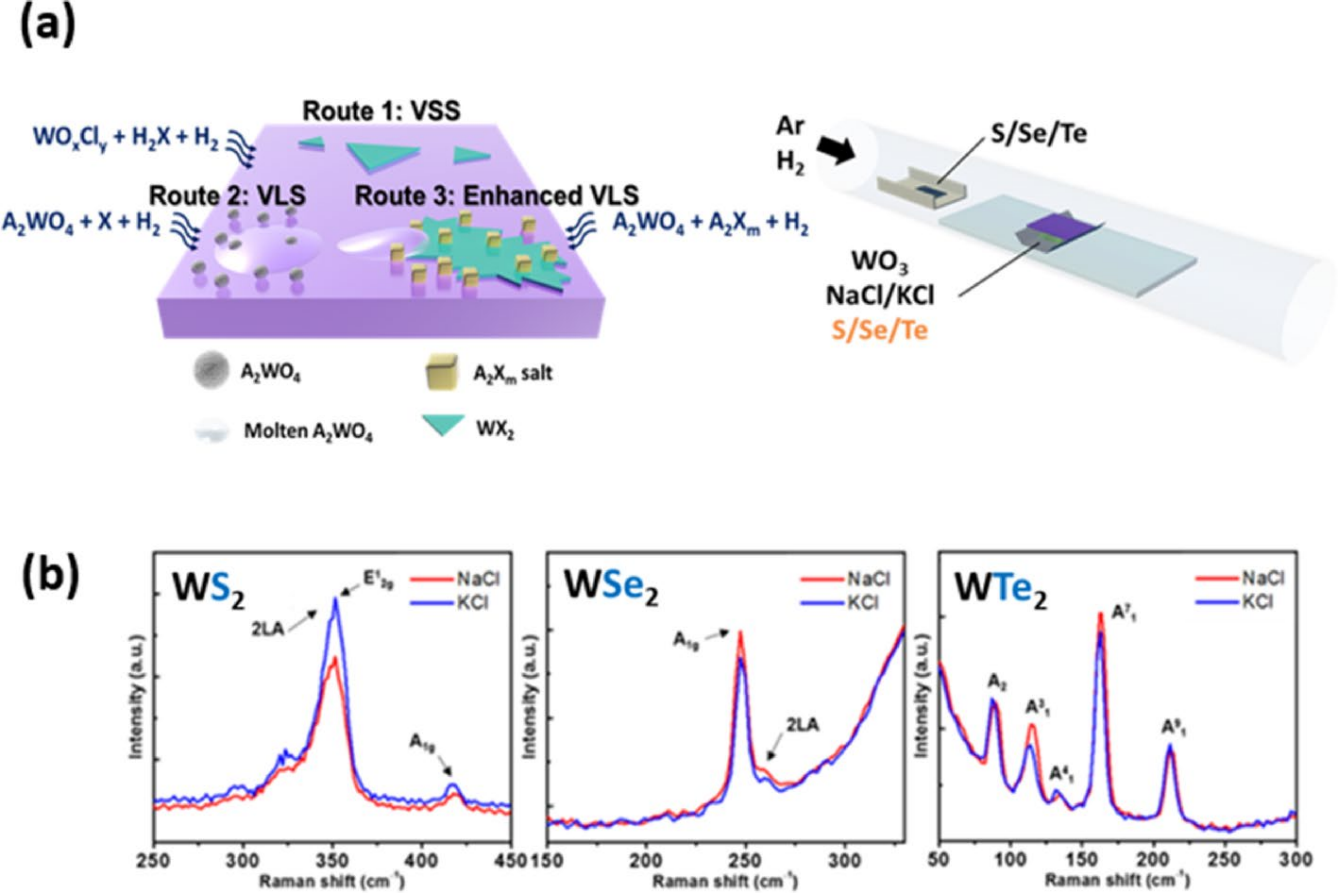
Alkali-Chalcogen-assisted growth. **a** Schematic illustration of the two representative growth regimes in CVD of the W-based TMD (WX_2_). **b** Raman spectra of the synthetic WX_2_ (X = S, Se, and Te)

Figure 1a illustrates the representative growth pathways enabled by promoter-assisted CVD for W-TMD. In reported studies, promoter-derived intermediate species play a key role in steering specific reaction mechanisms, thereby enabling the growth via two representative modes: VSS [1–5] and VLS [7–13]. In **Route 1** (VSS), gaseous tungsten oxychlorides (WOₓClᵧ), such as WOCl_2_, WO_2_Cl_2_, and WOCl_4_, are easily produced by the reaction of WO_3_ with alkali chlorides (AkCl) under reductive conditions. These intermediates subsequently react with hydrogen chalcogenides (H_2_X) to form crystalline WX_2_ via the VSS growth. The corresponding reactions are summarized in Eqs. (1) and (2):

1 $${\text{WO}}_{3} + {\text{AkCl}} + {\text{X}} + {\text{H}}_{2} \to {\text{WO}}_{{\text{x}}} {\text{Cl}}_{\gamma } + {\text{Ak}}_{2} {\text{WO}}_{4} + {\text{H}}_{2} {\text{X}}$$

2 $${\text{WO}}_{{\text{x}}} {\text{Cl}}_{{\text{y}}} + {\text{X}} + {\text{H}}_{2} {\text{X}} \to {\text{WX}}_{2} + {\text{HCl}} + {\text{H}}_{2} {\text{O}}$$

In **Route 2** (VLS), an excess amount of chalcogen (X = S, Se, or Te) reacts with the alkali chlorides (AkCl) to generate alkali chalcogenides (Ak_2_X_a_), as shown in Eq. (3). The formed Ak_2_X_a_ subsequently react with WO₃ to form alkali tungsten bronze (Ak_b_WO_3_) and alkali tungstates (Ak_2_WO_4_, such as Na_2_WO_4_ or K_2_WO_4_) under a hydrogen atmosphere, as detailed in Eq. (4).

3 $${\text{AkCl}} + {\text{X}} + {\text{H}}_{2} \to {\text{Ak}}_{2} {\text{X}}_{{\text{a}}} + {\text{HCl}}$$

4 $${\text{Ak}}_{2} {\text{X}}_{{\text{a}}} + {\text{WO}}_{3} + {\text{H}}_{2} \to {\text{Ak}}_{{\text{b}}} {\text{WO}}_{3} + {\text{Ak}}_{2} {\text{WO}}_{4} + {\text{H}}_{2} {\text{O}}$$

Given the relatively low melting points of alkali tungstates (Na_2_WO_4_: 698 °C; K_2_WO_4_: 921 °C) [34], these species can partially sublimate and diffuse to the substrate surface during heating. The molten Ak_2_WO_4_ serves as a liquid intermediate precursor, facilitating surface wetting [35, 36] and promoting subsequent reaction with Ak_2_Xₐ to yield WX_2_ films via a VLS growth, as expressed in Eq. (5):

5 $${\text{Ak}}_{2} {\text{X}}_{a} + {\text{Ak}}_{2} {\text{WO}}_{4} + {\text{H}}_{2} \to {\text{WX}}_{2} + {\text{Ak}}_{2} {\text{O}} + {\text{H}}_{2} {\text{O}}$$

In **Route 3**, an enhanced VLS growth is achieved by mixing excess chalcogen powders, WO_3_, and alkali halide promoters (NaCl or KCl). This approach enables the growth of continuous few-layer WX_2_ films (1.4 ~ 5.15 nm) with full coverage and an average sample size of approximately 1 cm × 1 cm, as determined from the optical/AFM images in Figure S2.

In Fig. 1b, the Raman spectra of the as-grown W-TMD are consistent with reported values of the characteristic vibrational modes [14, 37, 38]: For WSe_2_, the most intense peak corresponds to the A_1g_ mode at 247.3 cm^−1^. The Raman spectrum of WTe_2_ reveals five distinct modes located at 89.3 cm^−1^ (A_2_), 113.9 cm^−1^ (A^3^_1_), 133.6 cm^−1^ (A^4^_1_), 162.9 cm^−1^ (A^7^_1_), and 211.6 cm^−1^ (A^9^_1_). The improved film uniformity and areal coverage observed in this route reflect an enhanced VLS growth process, which benefits from a chalcogen-rich environment by forming stabilized Ak₂Xₐ intermediates (Ak = Na, K; X = S, Se, Te; a ≥ 1).

## Interfacial alkali cahalcognes

**Fig. 2:**
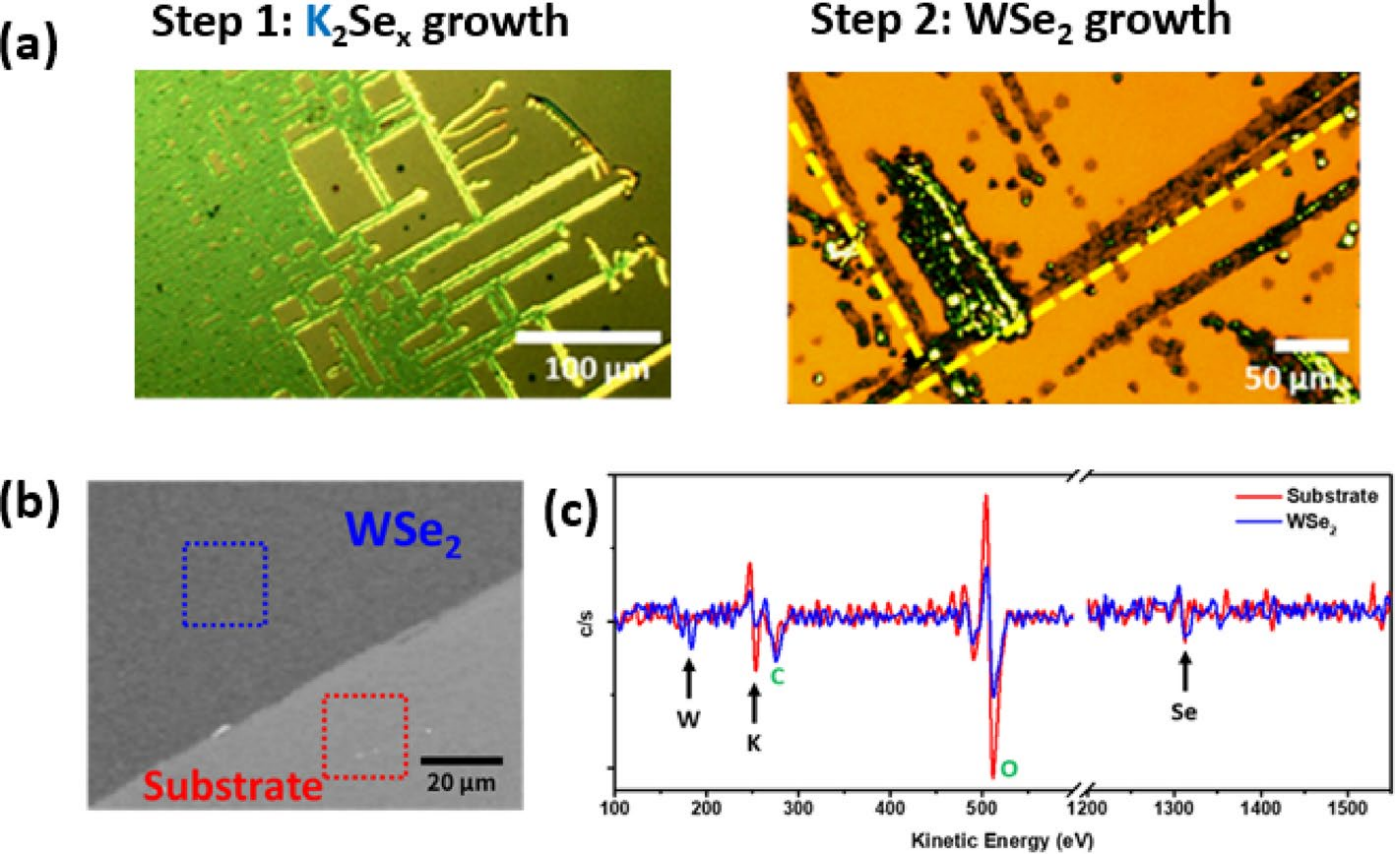
Verification of the interfacial alkali chalcogens. **a** Optical microscopy images of the two-step growth process, showing the formation of a salt-like K_2_Seₐ intermediate layer during Step 1, prior to the introduction of tungsten precursors. In Step 2, the WSe_2_ emerges upon the supply of W-based reactants. **b** SEM image of the WSe_2_ film after PDMS-assisted delamination, revealing a well-defined boundary between the remaining WSe_2_ and the exposed SiO_2_ substrate, serving as a contrast to highlight the interfacial region. **c** AES spectra acquired from the red (substrate) and blue (WSe_2_) regions marked in (**b**), showing clear compositional differences between the two areas

To further investigate the growth mechanism in Route 3 and verify the viability of synthesizing W-TMD from alkali chalcogenide, a two-step growth strategy was employed. In the first step, the alkali chalcogenides were deposited on SiO_2_/Si substrates by heating a mixture of alkali salts (NaCl or KCl) and chalcogen powders (S, Se, or Te) in the absence of any metal precursors. The deposition was conducted at 700 °C for 20 min. The residual chalcogen was reduced under a hydrogen atmosphere, yielding alkali polychalcogenides (Ak_2_Xₐ) [39]. In Fig. 2a, a dendritic shape of the potassium selenides (K_2_Seₐ) is formed on the substrate. In the second step, the substrate was positioned face-down over a tungsten crucible containing WO_3_ powders, followed by annealing at 700 °C under an Ar/H₂ atmosphere. Upon WO_3_ vaporization, partial decomposition of K_2_Seₐ occurred, leading to the formation of potassium tungstate (K_2_WO_4_) as a significant intermediate for VLS mode [7, 36, 40]. Residual alkali chalcogenide on the substrate subsequently reacted with molten K_2_WO_4_ via a VLS route, promoting the nucleation and growth of WSe_2_. Compared to conventional VLS mode, the use of alkali chalcogenide as promoter offers a lower energy barrier for reaction than direct reactions of vapor-phase chalcogen. Importantly, no chlorine was involved in the second step, supporting the hypothesis that alkali metal cations facilitate crystal growth independent of halide chemistry [40].

Alkali chalcogenides (Na_2_S or K_2_S) are expected to form at the interface between as-grown TMD flakes and the substrate during CVD when alkali salts (NaCl or KCl) are employed as growth promoters. To verify this hypothesis, a WSe_2_ synthesized via KCl-assisted growth was mechanically delaminated using a PDMS stamp to expose the interface. In Fig. 2b, a distinct boundary between the remaining WSe_2_ and the exposed substrate was observed, suggesting the presence of interfacial alkali chalcogenides. To further characterize its composition, Auger electron spectroscopy (AES) was performed (Figure S4**).** Figure 2c displays AES spectra acquired from the marked regions in Fig. 2b, revealing clear compositional contrast in the W NNN (184 eV) and K KLL (254 eV) transitions. Notably, in the exposed substrate region, only potassium and selenium signals—specifically the Se LMM (1312 eV) transition—were detected, consistent with the presence of residual potassium selenide (K_2_Se_x_) beneath the WSe_2_. These findings corroborate the presence of a potassium chalcogenide layer at the WSe_2_/substrate interface.

## Water-assisted transfer for ultraclean manipulation

**Fig. 3:**
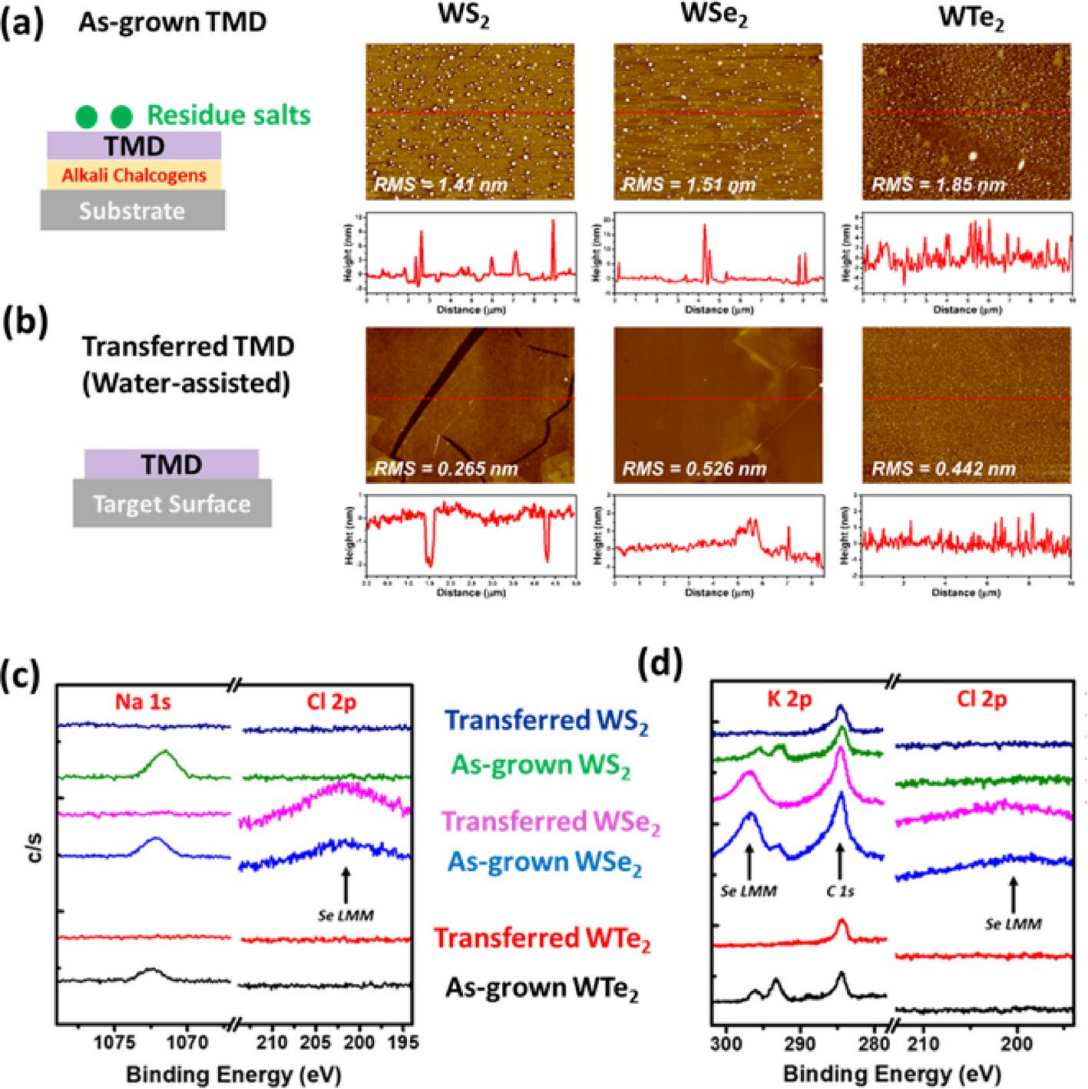
Water-assisted transfer of the as-grown films. Schematic illustration, AFM images and height of (**a**) the as-grown and **b** the transferred TMDs. **c** XPS spectra of Na 1*s* and Cl 2*p* core levels for NaCl-assisted WX_2_ films before and after the transfer. **d** XPS spectra of K 2*p* and Cl 2*p* core levels for KCl-assisted WX_2_ films before and after the transfer

Owing to the high water solubility of alkali chalcogenides (Na_2_S or K_2_S), a support-free transfer strategy was developed, enabling the direct manipulation of as-grown TMD onto target substrates via water-assisted delamination. Upon immersion in deionized water, capillary forces drove water infiltration at the film/substrate interface, promoting delamination through dissolution of the underlying alkali chalcogenide layer [24]. The released films were then gently transferred onto arbitrary substrates and dried under ambient conditions. A schematic and corresponding optical image of the process are shown in Fig. 3a and Figure S5, respectively. This method allows for the clean delamination and manipulation of synthetic TMDs facilitated by the interfacial water-soluble layer. As demonstrated in Figure S6, a PMMA film deposited on alkali chalcogenides was transferred using this approach. Notably, the process does not require any additional support layers, as the as-grown films exhibit sufficient mechanical integrity for direct handling. Raman spectroscopy (Figure S7) was used to evaluate the crystallinity of the transferred films. All samples displayed vibrational features consistent with those of the as-grown TMDs. A consistent blue shift of ~ 2–3 cm^−1^ was observed across all spectra. The shift in the A_1_g (out-of-plane) mode is attributed to electron doping likely induced by residual alkali species, in agreement with X-ray photoelectron spectroscopy (XPS) results (Figures S8 and S9). Meanwhile, the shift in the E_2_g (in-plane) mode is ascribed to strain relaxation following film release from the substrate. AFM was also employed to investigate the surface morphology before and after transfer. As-grown samples exhibited scattered salt particles with diameters around 500 nm and heights of approximately 15 nm. Consistent with the XPS and Raman results, these residues were no longer present after transfer, confirming the effective removal of the alkali chalcogenides. Compared to conventional PMMA-assisted transfer, a notable improvement in surface quality was observed in the transferred films. Although some cracks and folds were inevitably introduced during the manual handling, the overall crystalline quality remained well-preserved, and no detectable residues were observed.

To further examine the chemical configurations of the W-TMD films after the transfer process, XPS was conducted. (Figures S8, S9, 3c, and 3d) Notably, signals corresponding to alkali metals (Na and K) were absent in the transferred samples, and no detectable chlorine was observed, indicating that the alkali metals had reacted with chalcogen elements to form water-soluble compounds, which were subsequently removed during the transfer. More discussion is presented in SI-S11. The water-assisted transfer process of the synthetic W-TMD shows a robust performance.

## STM imaging of the transferred WTe_2_

**Fig. 4:**
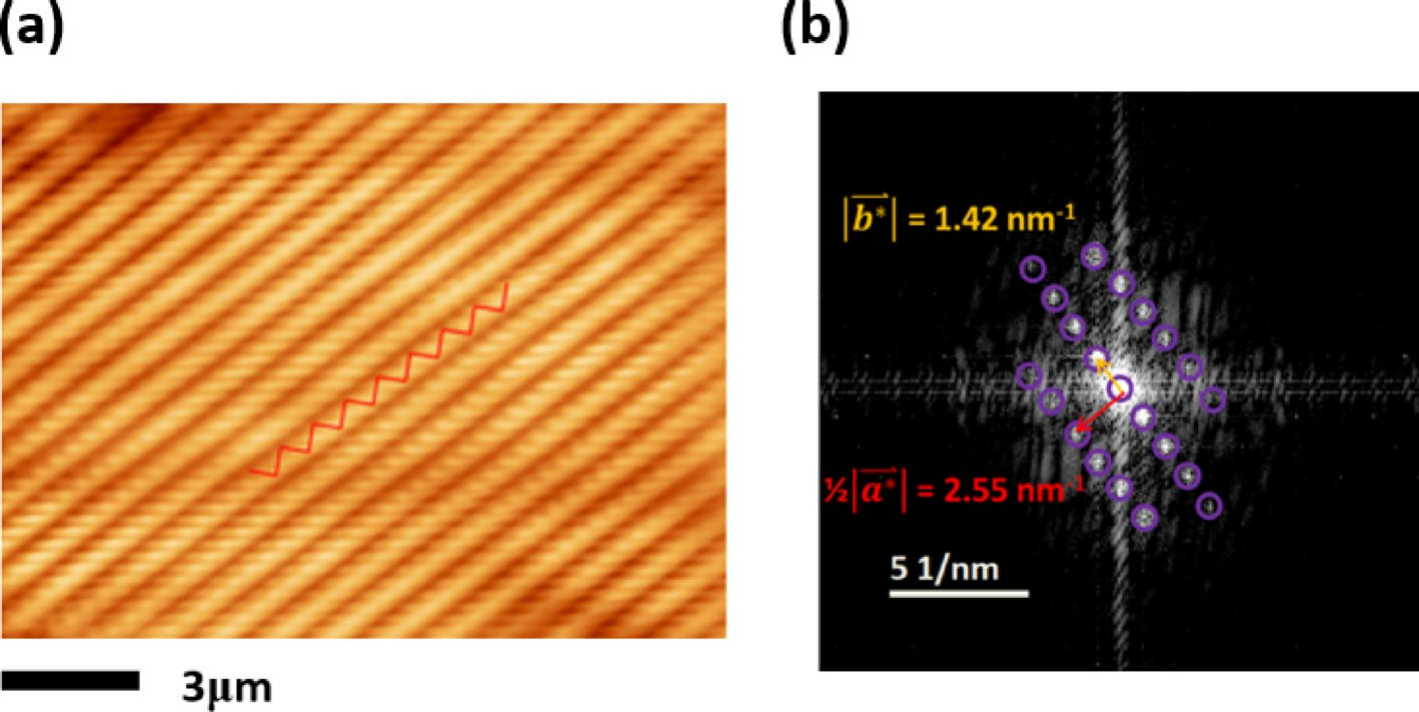
Atomically resolved STM imaging of the synthetic WTe_2_. **a** Constant-current scanning tunneling microscopy (STM) image of a transferred WTe_2_ film on highly ordered pyrolytic graphite (HOPG), showing a well-ordered atomic lattice. **b** Corresponding fast Fourier transform (FFT) image, with purple circles highlighting the characteristic Bragg diffraction vectors

To illustrate the ultraclean manipulation of the synthetic W-based TMD, scanning tunneling microscopy (STM) measurement of the transferred WTe_2_ is performed at room temperature and an ultrahigh vacuum (UHV). Before the measurement, as-grown WTe_2_ films are transferred to a highly ordered pyrolytic graphite (HOPG) substrate, and then annealed at 400 K for 12 h to remove surface adsorbs and contaminations. In Fig. 4a, atomically-resolved STM imaging of the WTe_2_ shows a perfect lattice arrangement of 1 T’ structure with parallel W-W zig-zag chains along the a-axis marked as a red zig-zag line. It manifests that the contribution of the metal charge density to the tunneling current is higher than that of chalcogens under a certain condition [41]. The corresponding FFT image in Fig. 4b well depicts the two-fold symmetry with a rectangular lattice. The reciprocal distances along the a and b axes are consistent with the result from the SAED pattern.

## Conclusion

We present an enhanced vapor–liquid–solid (VLS) growth strategy for the scalable synthesis of highly crystalline tungsten-based TMDs, enabled by the formation of alkali–chalcogen intermediate reactants. The salt-like interfacial alkali chalcogenides are synthesized and characterized via a two-step growth process. Atomically resolved STM imaging of transferred WTe_2_ confirms ultraclean manipulation, attributed to the presence of the salt-like alkali–chalcogen interfacial layer that enables support-free film delamination.

## Supplementary Information

Below is the link to the electronic supplementary material.

Supplementary Material 1.

## Data Availability

No datasets were generated or analysed during the current study.
