# Supplementary material for: Alkali chalcogenides-assisted vapor–liquid–solid growth of WX2 (X = S, Se, Te)
Source: Discov Nano. 2025 Sep 10;20(1):157. doi: 10.1186/s11671-025-04340-5 (PMC12423382; doi:10.1186/s11671-025-04340-5)
Supplement: Supplementary file 1 — Supplementary Material 1. [file 11671_2025_4340_MOESM1_ESM.docx]

Supporting Information

**Alkali Chalcogenides-Assisted Vapor–liquid–solid Growth of WX_2_ (X=S, Se, Te)**

Yi-Cheng Chiang^1^†, Po-Yen Liu^1^†, Erh-Chen Lin^1^†, Sheng-Hung Fan^1^, Chih-Chieh Hung^1^, Yu-Hiang Cheng^1^, Yi-Hsien Lee^1^*

^1^Department of Materials Science and Engineering, National Tsing Hua University, Hsinchu 30013, Taiwan

†These authors contribute equally to this work

*E-mail: yhlee.mse@mx.nthu.edu.tw

**Summary**

**S1. Experimental methods**

**S2. Comparison of the TMD Growth with excess chalcogen in the precursor mixtures**

**S3. Detailed reactions in the enhanced VLS growth with alkali chalcogenides**

**S4. Chemical composition of the pre-deposited alkali chalcogenides**

**S5. Water-assisted transfer of the synthetic W-TMD**

**S6. Water-assisted transfer of the coated PMMA films**

**S7. Raman spectra of the transferred W-TMD**

**S8. XPS of the as-grown and the transferred WX_2_ synthesized with NaCl promoter**

**S9. XPS of the as-grown and the transferred WX_2_ synthesized with KCl promoter**

**S10. XPS of the as-grown WTe_2_**

**S11. More details on the transferred W-TMD**

**S12. Reference**

**S1. Experimental methods**

***Promoter-assisted growth of the W-TMD***: The promoter-assisted growth of the TMD is studied by using a home-built chemical vapor deposition in ambient pressure (APCVD). A crucible containing the tungsten oxide precursors was placed at the center of the tube furnace, and a SiO_2_/Si substrate was face-down above the precursors. A mixture of 40 mg WO_3_ powders, 10 mg alkali salts (NaCl, KCl), and 10 mg chalcogen (S, Se, Te) powders was placed directly beneath the substrate. An additional ceramic crucible containing the chalcogen powders was positioned in the upstream zone of the furnace. To accommodate the different melting points, S, Se, and Te powders were placed in heating zones maintained at 180 °C, 280 °C, and 600 °C, respectively. The furnace was ramped to a reaction temperature of 700 °C over 15 minutes and held at the temperature for 5 minutes. Subsequently, the furnace cooled down to room temperature naturally. Throughout the process, argon and hydrogen gases were flowed at rates of 40 sccm and 15 sccm, respectively.

***Water-assisted transfer***: The as-grown TMD sample was immersed in de-ionized water at a low angle until the entire film delaminated and floated on the water surface. A clean target substrate was then used to retrieve the floating film, allowing it to gently adhere to the new substrate. The transferred films were subsequently dried in ambient air.

***Surface and optical characterizations***: The surface morphology of the as-grown tungsten dichalcogenides was examined using optical microscopy (OM, Olympus BX51TRF) and atomic force microscopy (AFM, Bruker ICON). Raman spectroscopy was conducted to identify vibrational modes by using the Raman system (ProTrusTech RAMaker) with a 532 nm excitation laser. The spectra were calibrated using the Raman peak of Si at 520.6 cm^-1^.

***Chemical and composition characterizations***: Chemical configurations of the synthetic TMD and the residual salts were analyzed by nano-Auger electron spectroscopy (AES, ULVAC-PHI PHI 700) and X-ray photoelectron spectroscopy (XPS, ULVAC-PHI PHI 5000 Versaprobe II). Both AES and XPS measurements were performed under ultra-high vacuum (UHV) conditions (~5 x 10^-9^ torr) with charge neutralization provided by an argon ion beam. AES was conducted using a 5 nA primary beam current at 10 kV beam energy. XPS measurements employed a monochromatic Al Kα X-ray source (1486.6 eV) with a spot size of 200 μm. Chemical compositions of alkali tungsten bronze powders were further characterized using field-emission scanning electron microscopy (FE-SEM, JEOL JSM-7610F) equipped with an energy-dispersive X-ray spectroscopy (EDX) detector (Oxford MAX 50).

***STM measurement:*** The STM samples were prepared with the conductive HOPG substrate by using water-assisted transfer of the WTe2 sample synthesized in the promoter-assisted CVD. The samples were loaded into an ultra-high vacuum chamber for the degassing process at a temperature of ~140℃ with a working pressure lower than 1×10^−9^ mbar. STM imaging of the transferred samples is performed on a commercial STM (Omicron VT) at room temperature with a chemically etched tungsten tip. All the STM images are acquired under constant current mode and processed with the WSxM software.

**S2. Comparison of the TMD Growth with excess chalcogen in the precursor mixtures**

**
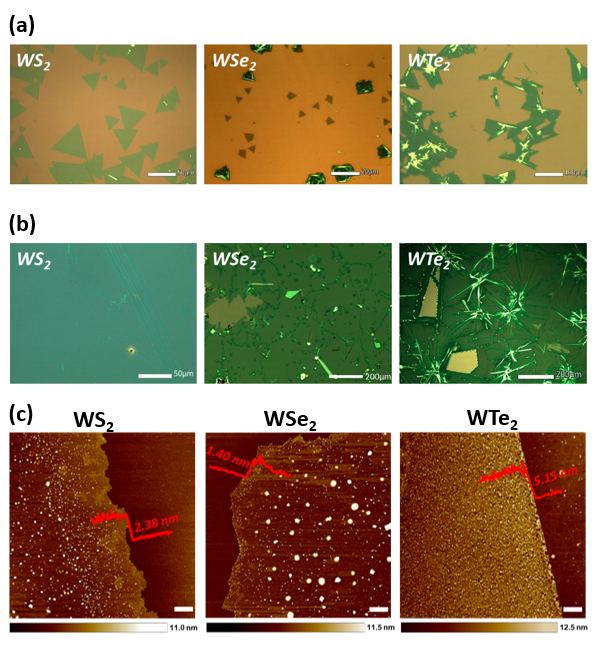
**

**Figure S1.** OM images of as-grown TMDs (a) without and (b) with additional chalcogen powders in the NaCl–WO₃ precursor mixture. (c) AFM images of as-grown TMD with the precursor mixture. The insets are the corresponding height profiles. Scale bars are 1 µm.

**S3. Detailed reactions in the VLS growth with alkali chalcogenides**

Previous studies have confirmed the formation of sodium tungstate (Na_2_WO_4_) and sodium molybdate (Na_2_MoO_4_) in NaCl-assisted CVD systems.^[1–4]^ Furthermore, the presence of chalcogen sources can promote the partial reduction of WO_3_ to substoichiometric suboxides (WO_3-x_), which subsequently undergo chalcogenization to form WO_c_X_d_ intermediates on the substrate surface.^[5,6]^ Both alkali tungstate (Na_2_WO_4_ or K_2_WO_4_) and tungsten bronze (Ak_b_WO_3_) are expected to coexist during the VLS growth.

Notably, the relatively low melting point of alkali tungstates compared to tungsten bronzes enables their sublimation under CVD conditions, serving as a volatile tungsten source. Optical microscopy images of post-reacted WO₃ powders with NaCl and KCl (Figure S3 b,c) reveal a variety of colors characteristic of tungsten bronzes. These color variations correlate with the value of *b* in Ak_b_WO_3_, ranging from dark blue (b ≈ 0.2–0.4), through red (b ≈ 0.6–0.8), to yellow (b > 0.8), as previously reported.^[7,8]^ Energy-dispersive X-ray spectroscopy (EDX) analysis (Figure S1g) confirms that the dominant compositions of the resulting bronzes are Na_0.7_WO_3_ and K_0.28_WO_3_ for NaCl- and KCl-assisted samples, respectively. These stoichiometries were determined from individual crystal regions and agree with the observed optical characteristics.


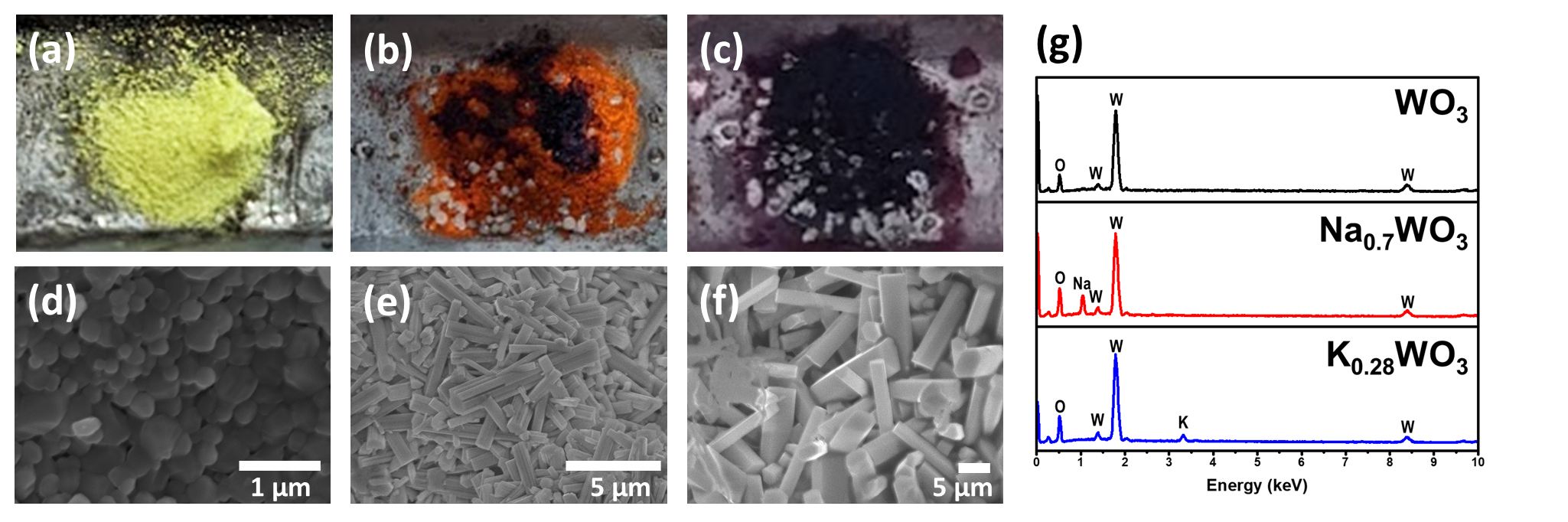


**Figure S2.** Characterization of alkali tungsten bronze powders. Optical image of (a) pristine WO_3_ powders, (b) as-reacted WO_3_ powders with NaCl, and (c) as-reacted WO_3_ powders with KCl. SEM image of (d) pristine WO_3_ powders, (e) as-reacted WO_3_ powders with NaCl, and (f) as-reacted WO_3_ powders with KCl. (g) EDX spectrum taken from (d-f).

**S4. Chemical composition of the pre-deposited alkali chalcogenides**

To confirm the elemental composition of the deposited alkali chalcogenides on the substrates, Auger electron spectroscopy (AES) was employed. AES was selected over X-ray photoelectron spectroscopy (XPS) due to its superior spatial resolution, which enables localized probing of specific regions and minimizes interference from unreacted alkali salts present on the surface. The recorded Auger spectra exhibited characteristic peaks corresponding to the constituent elements of the alkali chalcogenides. Importantly, no chlorine signals (Cl LMM, ~184 eV) were detected, indicating that the observed alkali metal signals originated from chalcogenide compounds rather than residual chloride salts. Additionally, the peaks observed at approximately 275 eV and 512 eV were assigned to the C KLL and O KLL transitions, respectively. After the deposition of alkali chalcogenides on the substrates, we used AES to verify the elementary composition. The reason why we chose AES rather than XPS was the former provided excellent spatial resolution to probe ideal regions. Hence, we could eliminate the concern of detecting unreacted alkali salts on the surface. In general, the Auger peaks were completely corresponding to the typical elements in alkali chalcogenides. No detected chlorine (184 eV) indicated the signals of alkali elements were from chalcogenides instead of original chloride salts. Note that the peaks at 275 and 512 eV in AES spectra are attributed to C KLL and O KLL transition.


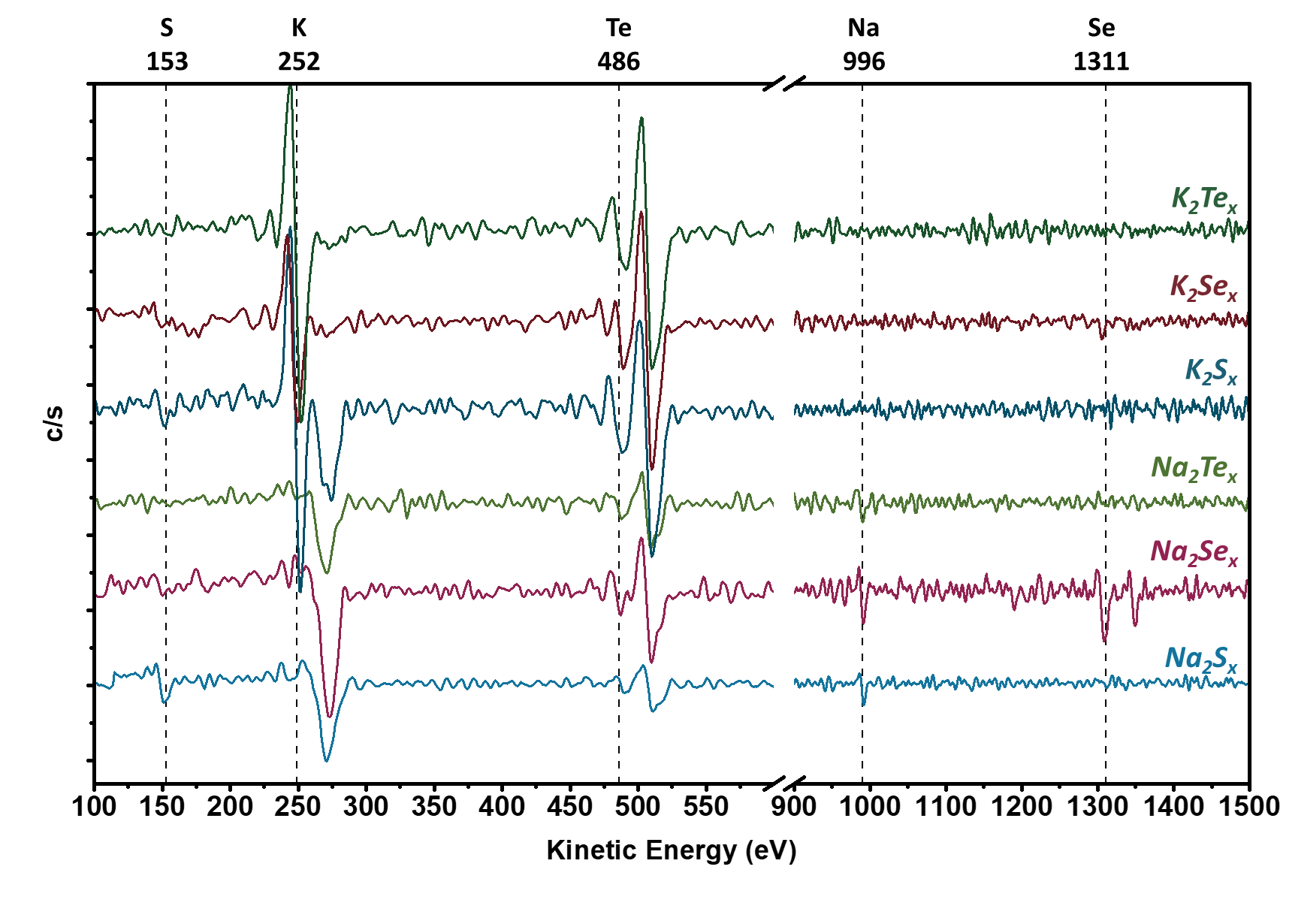


**Figure S3. Verification of the as-grown alkali chalcogenides:** AES spectra of the pre-deposited alkali chalcogenides.

**S5. Water-assisted transfer of the synthetic W-TMD**


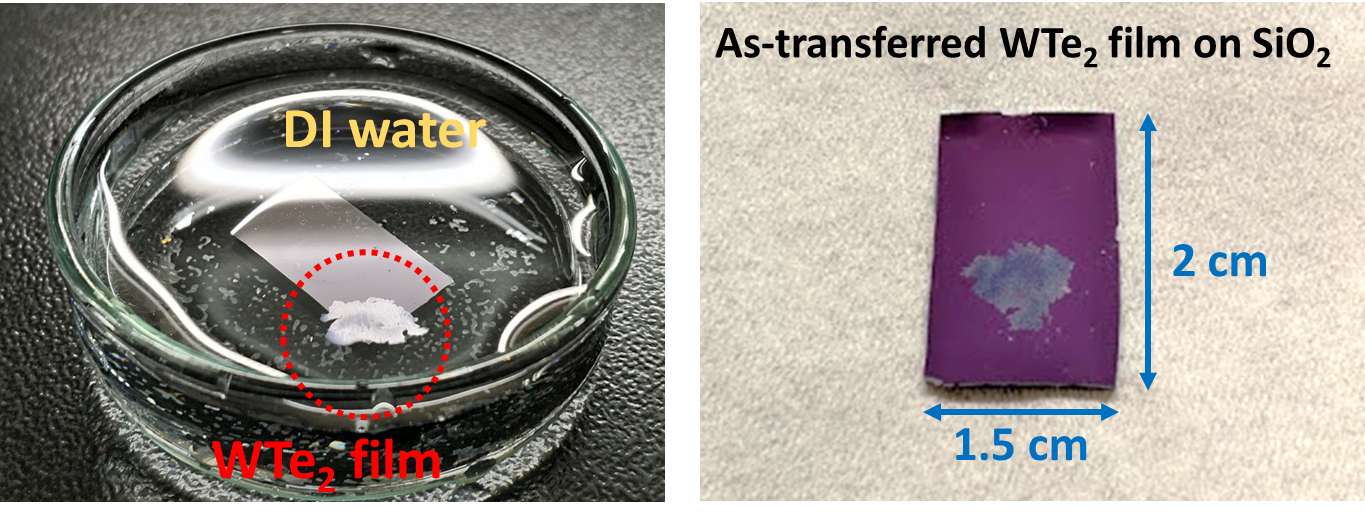


**Figure S4.** A thick WTe_2_ (thickness of~120 nm) is adopted to better illustrate the water-assisted transfer process

**S6. Water-assisted transfer of the coated PMMA films**


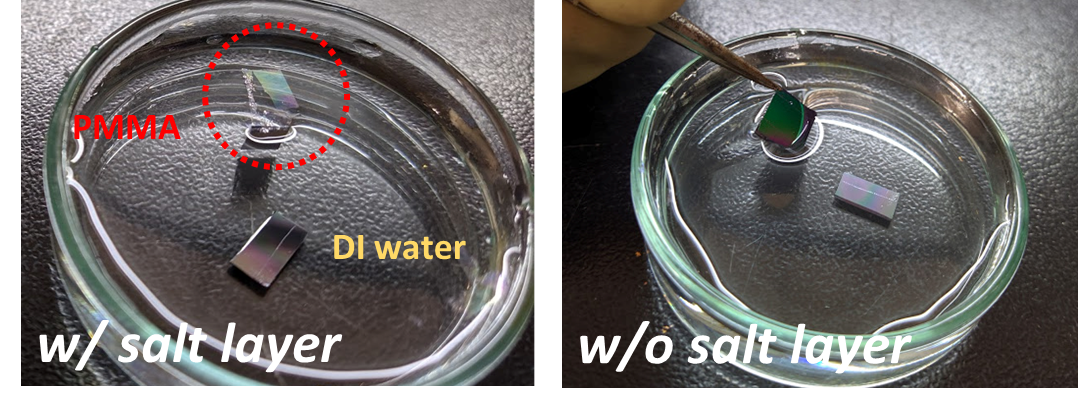


**Figure S5.** Comparison of the water-assisted transfer of a PMMA-coated layer with and without an interfacial salt-like layer.

**S7. Raman spectra of the transferred W-TMD**


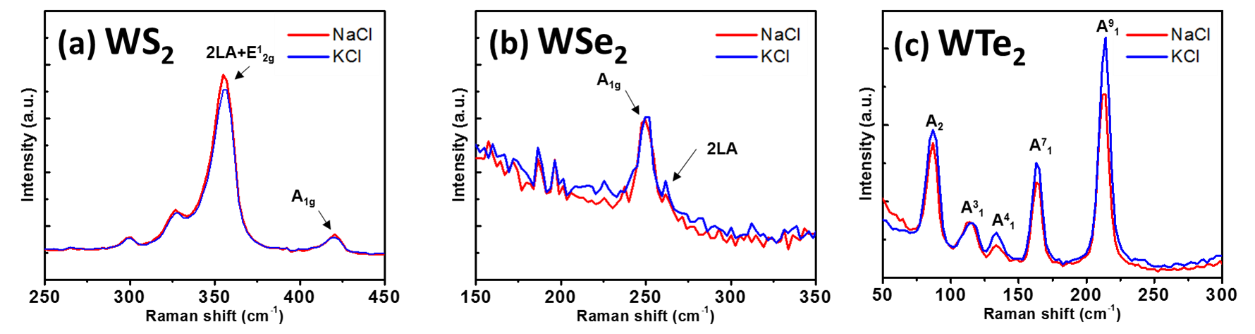


**Figure S6.** Raman spectra of the transferred (a) WS_2_, (b) WSe_2_, and (c) WTe_2_. Red and blue lines indicate the promters used in the synthesis.

**S8. XPS of the as-grown and the transferred WX_2_ synthesized with NaCl promoter**


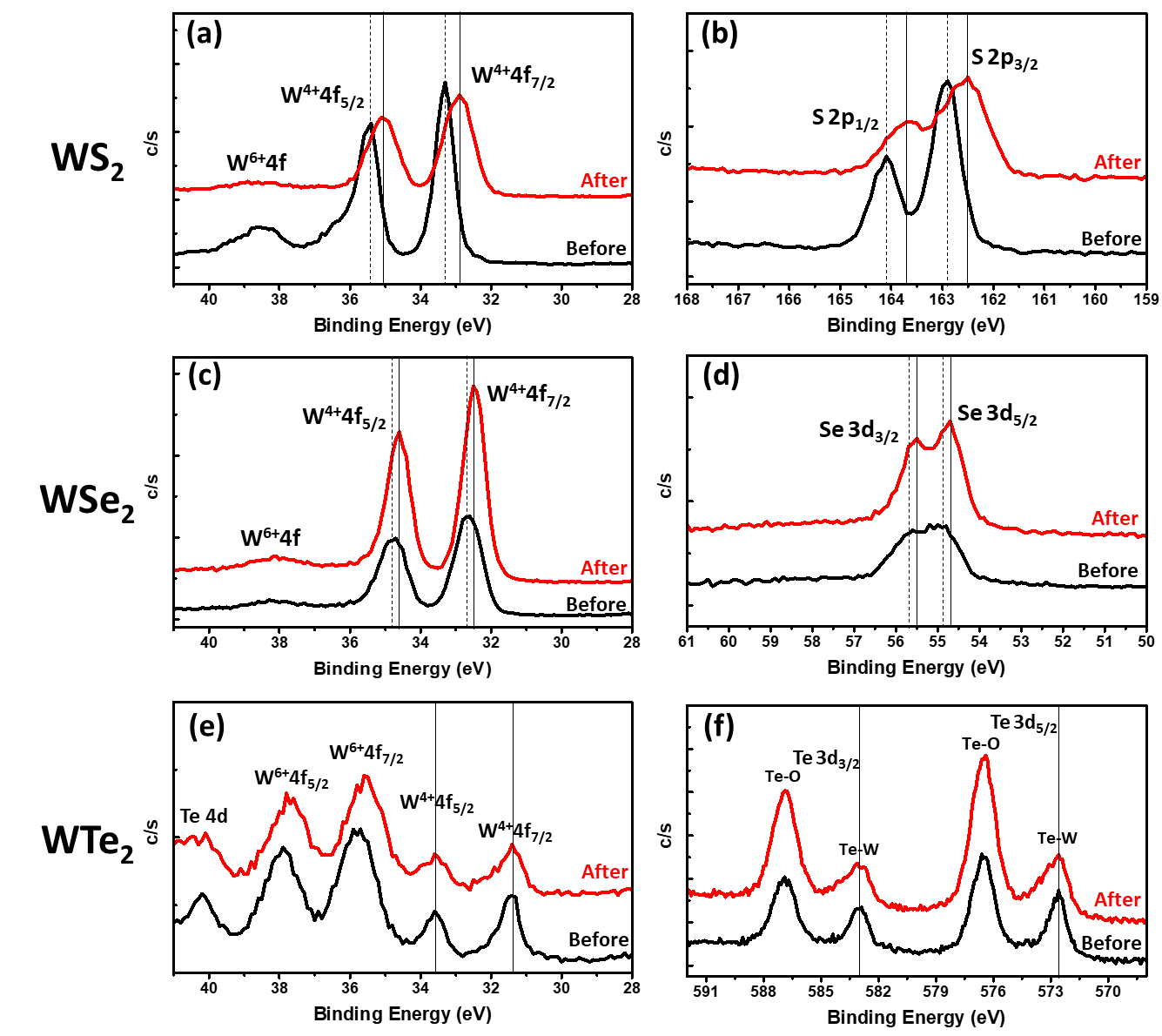


**Figure S7. XPS spectra** **of NaCl-assisted WX_2_ films before and after the transfer**

(a, b) W 4f and S 2p orbits of WS_2_. (c, d) W 4f and Se 3d orbits of WSe_2_.

**S9. XPS of the as-grown and the transferred WX_2_ synthesized with KCl promoter**


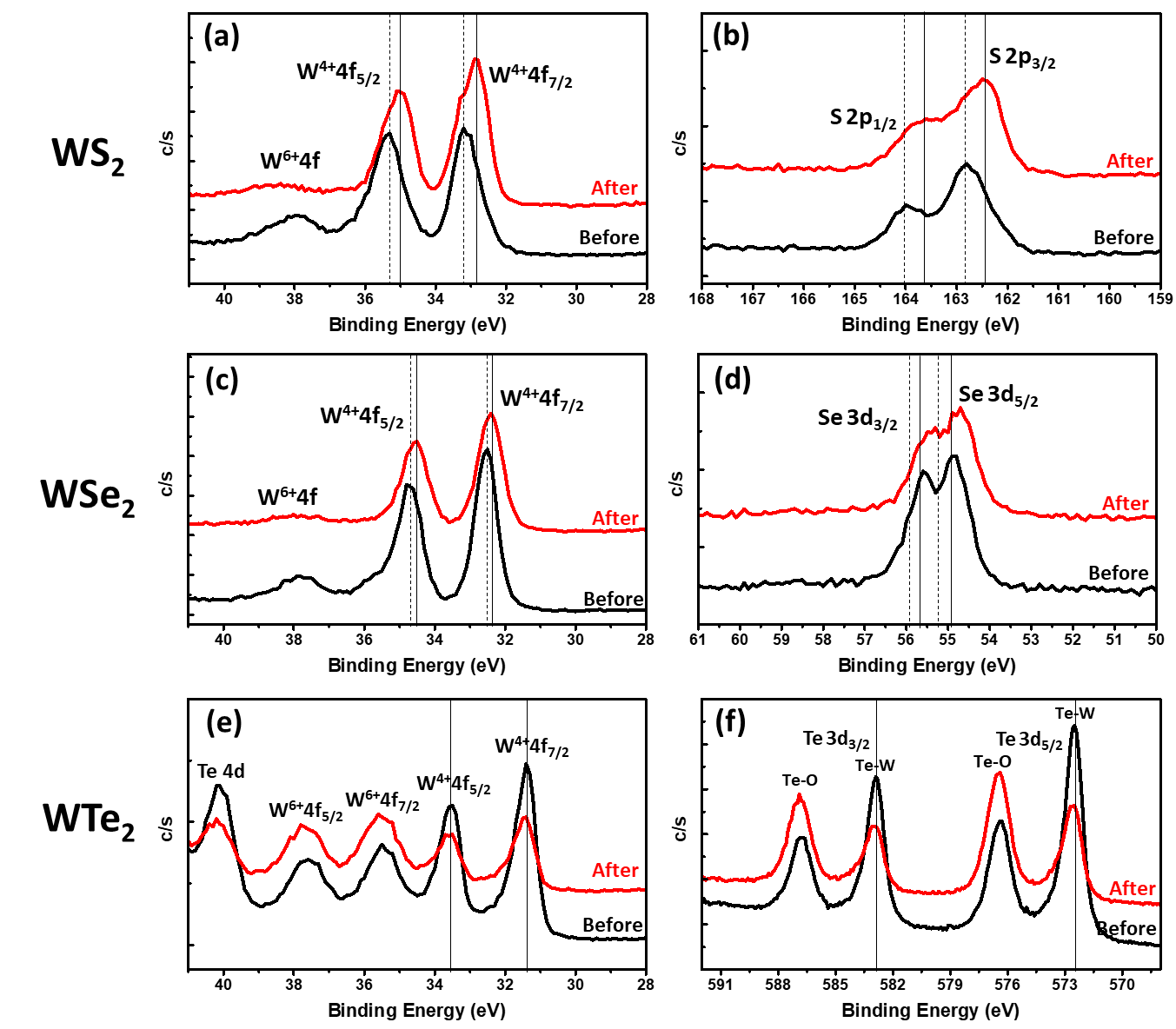


**Figure S8. XPS spectra of KCl-assisted WX_2_ films before and after the transfer**

(a, b) W 4f and S 2p orbits of WS_2_. (c, d) W 4f and Se 3d orbits of WSe_2_.

**S10. XPS of the as-grown WTe_2_**

**
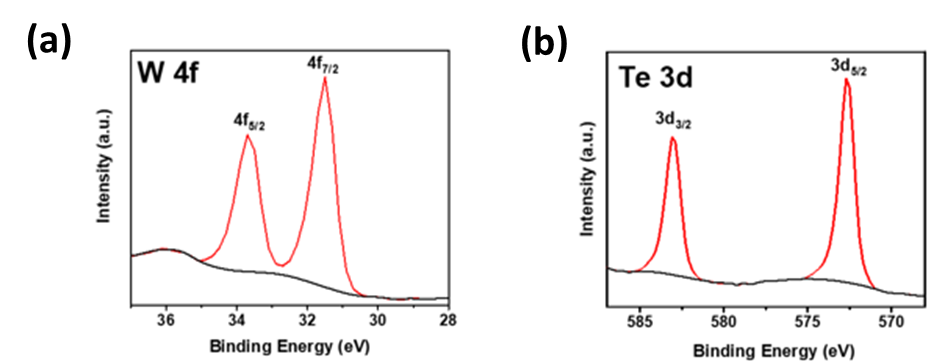
**

**Figure S9.** XPS spectra of the as-grown WTe_2_ films: W 4f and Te 3d orbits of WTe_2_.

**S10. More details on the transferred W-TMD**

For the NaCl-assisted series, the Na 1s binding energies were observed at 1072.4, 1072.2, and 1071.5 eV for WS_2_, WSe_2_, and WTe_2_, respectively. In the KCl-assisted series, the K 2p₃/₂ peaks appeared at 293.3, 292.9, and 292.5 eV for the corresponding dichalcogenides. The systematic downshift in binding energies from S to Te is attributed to the decreasing electronegativity of the chalcogen elements (S: ~2.58, Se: ~2.55, Te: ~2.12), which alters the electron distribution in the alkali–chalcogen bonds. In the spectra of WSe_2_ samples, additional features at 201.3 and 296.9 eV correspond to the Se L_3_M_4,5_M_4,5_ and L_3_M_2,3_M_4,5_ Auger transitions, respectively.^[9]^

**S11. Reference**

1. Rasouli, H.R., et al., Real-time optical observation and control of atomically thin transition metal dichalcogenide synthesis. Nanoscale, 2019. 11(15): p. 7317-7323.
2. Li, S., et al., Vapour-liquid-solid growth of monolayer MoS2 nanoribbons. Nat Mater, 2018. 17(6): p. 535-542.
3. Johnson, D.A., et al., Purification of molybdenum: volatilisation processes using MoO3. Polyhedron, 1982. 1(5): p. 479-482.
4. Shinde, S.M., et al., Stacking-controllable interlayer coupling and symmetric configuration of multilayered MoS2. NPG Asia Materials, 2018. 10.
5. Zafar, A., et al., Sulfur-Mastery: Precise Synthesis of 2D Transition Metal Dichalcogenides. Advanced Functional Materials, 2019. 29(27).
6. Cong, C.X., et al., Synthesis and Optical Properties of Large-Area Single-Crystalline 2D Semiconductor WS2 Monolayer from Chemical Vapor Deposition. Advanced Optical Materials, 2014. 2(2): p. 131-136.
7. Dickens, P.G. and M.S. Whittingham, The tungsten bronzes and related compounds. Quarterly Reviews, Chemical Society, 1968. 22(1): p. 30-44.
8. Mann, M., G.E. Shter, and G.S. Grader, Deposition of inorganic bronze coatings over ceramic foams. Journal of Materials Research, 2005. 20(5): p. 1207-1215.
9. Shallenberger, J. R. 2D tungsten diselenide analyzed by XPS. *Surface Science Spectra*, **2018**, *25*(1).
